# The EVITA framework for evidence-based mental health policy agenda setting in low- and middle-income countries

**DOI:** 10.1093/heapol/czz179

**Published:** 2020-02-10

**Authors:** Nicole Votruba, Jonathan Grant, Graham Thornicroft

**Affiliations:** 1 Centre for Global Mental Health, Health Service and Population Research Department, Institute of Psychiatry, Psychology & Neuroscience (IoPPN), King's College London, De Crespigny Park, Denmark Hill, London SE5 8AF, UK; 2 Centre for Implementation Science, Health Service and Population Research Department, Institute of Psychiatry, Psychology & Neuroscience (IoPPN), King's College London, De Crespigny Park, Denmark Hill, London SE5 8AF, UK; 3 Policy Institute at King’s, King’s College London, 1st Floor, Virginia Woolf Building, 22 Kingsway, London WC2B 6LE, UK

**Keywords:** Knowledge translation and exchange, evidence-informed policymaking, agenda setting, evidence uptake, research impact, evidence-based policymaking, research evidence, mental health, low- and middle-income countries, framework

## Abstract

The burden of mental illness is excessive, but many countries lack evidence-based policies to improve practice. Mental health research evidence translation into policymaking is a ‘wicked problem’, often failing despite a robust evidence base. In a recent systematic review, we identified a gap in frameworks on agenda setting and actionability, and pragmatic, effective tools to guide action to link research and policy are needed. Responding to this gap, we developed the new EVITA 1.1 (EVIdence To Agenda setting) conceptual framework for mental health research–policy interrelationships in low- and middle-income countries (LMICs). We (1) drafted a provisional framework (EVITA 1.0); (2) validated it for specific applicability to mental health; (3) conducted expert in-depth interviews to (a) validate components and mechanisms and (b) assess intelligibility, functionality, relevance, applicability and effectiveness. To guide interview validation, we developed a simple evaluation framework. (4) Using deductive framework analysis, we coded and identified themes and finalized the framework (EVITA 1.1). Theoretical agenda-setting elements were added, as targeting the policy agenda-setting stage was found to lead to greater policy traction. The framework was validated through expert in-depth interviews (*n* = 13) and revised. EVITA 1.1 consists of six core components [advocacy coalitions, (en)actors, evidence generators, external influences, intermediaries and political context] and four mechanisms (capacity, catalysts, communication/relationship/partnership building and framing). EVITA 1.1 is novel and unique because it very specifically addresses the mental health research–policy process in LMICs and includes policy agenda setting as a novel, effective mechanism. Based on a thorough methodology, and through its specific design and mechanisms, EVITA has the potential to improve the challenging process of research evidence translation into policy and practice in LMICs and to increase the engagement and capacity of mental health researchers, policy agencies/planners, think tanks, NGOs and others within the mental health research–policy interface. Next, EVITA 1.1 will be empirically tested in a case study.

**Key Messages**

- EVITA 1.1 is a novel, unique framework specifically designed to increase mental health research–policy traction on the policy agenda of low- and middle-income countries (LMICs). The links for policy agenda setting in mental health are not yet fully understood. In a novel approach, EVITA 1.1 combines knowledge translation and evidence-based policymaking with the effective mechanism of policy agenda setting.
- The new EVITA 1.1 framework is validated through two specifically designed frameworks for mental health-specific factors in LMICs and mental health policy issue priority setting.
- The new EVITA 1.1 framework aims to support and assist action for mental health research evidence and policymaking processes and interrelationships and to increase the engagement and capacity of mental health researchers, health policy agencies and planners, think tanks, NGOs and others working in the mental health policy interface.
- Based on a thorough methodology, and through its specific design and mechanisms, EVITA has the potential to improve the challenging process of research evidence translation into policy and practice in LMICs. To increase its’ actionability, the EVITA 1.1 framework will be empirically tested and validated.

## Introduction

Better and more use of health research in policymaking can save lives, reduce poverty and improve economic performance in low- and middle-income countries (LMICs) (Sutcliffe and Court, 2006). However, difficulties in the translation and uptake of research in policy have been coined as the ‘research–policy gap’ (Oliver *et al.*, 2014). This process is even more exacerbated for mental health, which can be considered a challenging policy issue, particularly in LMICs (Hyman *et al.*, 2006). Research evidence shows that the global burden for mental illness is excessive (Whiteford *et al.*, 2013; Vigo *et al.*, 2016) and that, in LMICs, up to 90% of people with mental health problems do not receive treatment (Demyttenaere *et al.*, 2004). Policies are a first step to change practice, by implementing, improving and scaling up mental health systems and services (Rathod *et al.*, 2017), but in many LMICs, such policies are missing and mental health is not on the policy agenda in the first place (Omar *et al.*, 2010; The Mental Health and Poverty Project, 2010).

Health policy research suggests that a specific focus on policy agenda setting can help to improve knowledge translation and increase the uptake and impact of research in policy (Hanney *et al.*, 2003; Sumner *et al.*, 2011). Few studies are aiming at improving the use of mental health research (Williamson *et al.*, 2015; Petek *et al.*, 2017), and while frameworks can help guide and support this process (Syed *et al*., 2013), we found that to date, no theoretical approach provides aims at increasing the uptake of mental health research on the policy agenda in LMICs and to give strategic guidance (Votruba *et al.*, 2018). Responding to this gap, this article presents the development and validation of the EVITA 1.1 (EVIdence To Agenda setting) framework, which applies an agenda-setting focus and specific mechanisms to increase the impact of mental health research in policy.

### Mental health is a special, challenging policy issue in LMICs

Mental health is globally neglected as a policy issue (Lund, 2018), and in LMIC settings, it faces specific, aggravating challenges (Saraceno *et al.*, 2007). Many factors have been identified to act as barriers to the uptake and use of mental health research on the policy agenda and for making it a special and unique policy case (Hornby and Perera, 2002; Bird *et al.*, 2011; Mackenzie, 2014; Howell *et al.*, 2017). First, mental health differs from other health policy issues by being a wide field with diverse conditions, highly individualized treatments and priorities, treatment and care globally being contested (Hyman *et al.*, 2006). In addition, cultural differences exist how mental illness is defined (Sartorius, 1988), how and when social behaviour is perceived as normal or deviant and what meanings, religious or spiritual explanations are assigned to it (Cheng, 2001). Different international classification systems add to the unclarity and conceptual controversies (Stein *et al.*, 2013). Furthermore, mental illness is often linked to other comorbidities and wider socioeconomic implications (Lund *et al.*, 2011; 2018; Cohen, 2017).

This challenge is also reflected in a lack of local LMIC data and cost evaluations (Bloom *et al.*, 2011), while literacy and capacity gaps persist on scaling up and cross-sectoral integration (Hyman *et al.*, 2006; Jamison *et al.*, 2006; Eaton *et al.*, 2011). In LMICs often challenging economic situations are linked to little local in-country research, low numbers of experts and other competing critical health and policy issues (Sutcliffe and Court, 2006; Razzouk *et al.*, 2010; Siriwardhana *et al.*, 2011). Social determinants, such as inequality, political instability, poverty, natural disasters, conflict, violence and high crime rates, exist and impact negatively on mental health and hinder implementation of systems and services (World Health Organization and Calouste Gulbenkian Foundation, 2014). And limited political, academic and media capacity and attention add to perpetuating the large research evidence gap to policy (Saraceno *et al.*, 2007; Malla *et al.*, 2018). Improving mental health systems, policies and care often requires policy solutions outside the mental health field, which means addressing cross-links with other health- and socioeconomic issues and co-creation with these research areas (Cohen, 2017).

Another major challenge is that mental illness is associated with high levels of stigma (Semrau *et al.*, 2015), but compared with other stigmatized, infectious diseases, such as human immunodeficiency virus (HIV)/acquired immune deficiency syndrome (AIDS), and fatal other NCDs, such as cardiovascular diseases, mental disorders, lack policy attention because the focus remains on early deaths, rather than lives lived with disabilities (Prince *et al.*, 2007). Although suicide contributes largely to the global burden of disease, it is usually not categorized under the effects of mental illnesses (Vigo *et al.*, 2016). As a result, mental health faces continuing low national financial investment, policy priority and media attention, and insufficient international commitment and engagement (Eaton *et al.*, 2011). Linked to the pervasive stigma, the agency of service users is still largely missing, compared with other health conditions such as HIV, where people affected and their families are protesting and demanding their rights (Burns, 2010). Advocacy and effective global networks are just about to rise (Mackenzie, 2014).

### The agenda-setting gap in mental health research and policy interrelationships

For these reasons, mental health remains a low policy priority in LMICs, as policymakers are seeing mental health as a ‘charity case with no return on investment’, despite better evidence (Saraceno *et al.*, 2007). However, little has been investigated to identify specific underlying causes and which theoretical approaches can be helpful to improve research uptake on the policy agenda and priority setting in LMICs (Bird *et al.*, 2011). Understanding research evidence and policy interrelationships is a critical first step for increasing research impact in policy (Oliver *et al.*, 2014) and, instead of the general policy process, specifically targeting the policy agenda stage, can be a key dimension for policy impact of evidence (Lavis *et al.*, 2002; Jones and Villar, 2008; Ogbe *et al.*, 2018). However, in a recent systematic review of theories and frameworks on mental health research evidence and policy interrelationships in LMICs, we found no framework targeted the policy agenda (Votruba *et al.*, 2018).

### What are research and policy interrelationships?

Policymaking is messy (Lindblom, 1959), and so are interrelationships between research and policy. Different terminologies and concepts refer to the translation, uptake and exchanges of research and policymaking, such as knowledge exchange, integrated knowledge translation or evidence uptake (Graham *et al.*, 2006; McKibbon *et al.*, 2010; Greenhalgh and Wieringa, 2011). In this article, we use the term ‘research and policy interrelationships’ to refer to all interactions and activities occurring and supporting understanding, communication and connection of scientific research knowledge and policy processes, including knowledge translation and exchange, or evidence-based policymaking. We have elaborated on the different understandings of research and policy interrelationships also in an earlier study (Votruba *et al.*, 2018). We understand a ‘researcher’ as scientific researcher, working in an academic, university or other research environment. We understand a ‘policymaker’ as someone who drafts, designs or contributes to (mental) health policy documents or programmes, or who informs, makes or contributes to policy decisions about (mental) health services, programmes and budgeting (Haynes *et al*., 2015; Redman *et al.*, 2015). For ease of presenting a conceptual model, these roles are separated, but they may overlap and change depending on time, issue and context.

### Why does the new EVITA 1.1 framework focus on policy agenda setting?

Research translation and uptake into policymaking is a ‘wicked problem’, and traditional methods have failed to provide explanation and improvement for mental health (Churchman, 1967; Hannigan and Coffey, 2011). Health policy research finds that researchers targeting the policy agenda setting stage will improve the uptake of research findings in policy (Ogbe *et al.*, 2018) and suggest that this will lead to greater policy traction for mental health research (Hanney *et al.*, 2003). The proposed EVITA 1.1 framework uses a focus on agenda setting as a mechanism for strengthening knowledge translation, research uptake and evidence-based policymaking in LMICs. So far, to our knowledge, no theoretical framework has applied an agenda setting focus to improve research–policy interrelationships in mental health in LMICs.

General conceptual approaches for policy agenda setting exist (Reich, 1995; Baumgartner *et al.*, 2006; True *et al*., 2007;Real-Dato, 2009; Kingdon, 2014; Fisher *et al.*, 2015), however the process remains complex and difficult to predict, as to when, why and what for, certain research gains public and policy attention, manages to mobilize resources and policy traction, while other research does not (Weiss, 1979; Hanney *et al.*, 2003). Policy agenda setting can involve multiple stakeholders from policy, research, media and society. A critical challenge is to facilitate behaviours that encourage policymakers to access research more systematically, and for research to better align with policy needs (Hanney *et al.*, 2003).

### A new definition of policy agenda setting

We define agenda setting to be the policy pre-decision process when a problem is identified, defined and prioritized, gains and maintains attention of policymakers, and eventually and becomes a policy priority. In an idealized process model, policy agenda setting partly overlaps with policy formation but occurs before policy decision-making and implementation (Baumgartner *et al.*, 2006; Nutley *et al.*, 2007; Kingdon, 2014; Votruba *et al.*, 2018; see Supplementary data 1 Simplified evidence–policy–practice model).

### Aims

The primary aim of this paper is to describe the development and validation through in-depth interviews of the action framework EVITA 1.1.

EVITA 1.1 is a framework for research EVIdence To Agenda setting. It focuses exclusively on research evidence and its interrelations with policymaking for mental health in LMICs, and targets policymaking at the agenda setting stage. Specifically, the EVITA 1.1 framework addresses the problem how to get mental health on the policy agenda for the first time/when it is first defined/or paid attention to. The framework aims to be ‘actionable’, which we define as ‘providing conceptual clarity, having a clear purpose, being able to explain how individuals move from intention to actual behaviour change, and useful to develop and test interventions’ (Votruba *et al.*, 2018). The aim of EVITA is to facilitate, analyse and guide mental health research and policy interrelationships, with the intention to serve as a ‘pragmatic, predictive and effective tool’ (Redman *et al.*, 2015) for improving research and policy exchange and enhance research impact on the policy agenda. EVITA primarily targets researchers to increase their capacity and engagement with policy. Other potential users could be individuals and organizations working in the mental health research–policy ecosystem, such as policymakers, health policy agencies and planners.

A secondary aim of this article is to validate the EVITA framework for mental health criteria. A tertiary aim of this article is to develop a simple framework to assess conceptual frameworks.

## Methods

**Figure 1:**
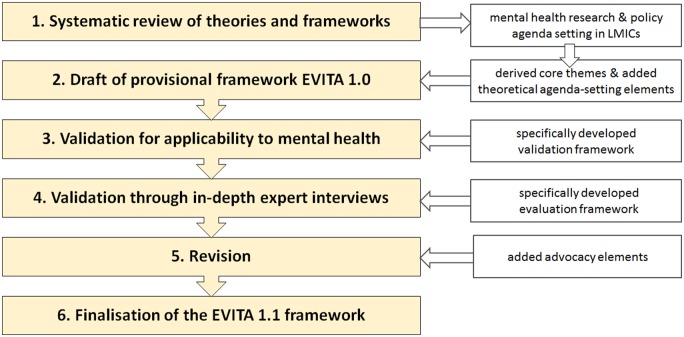
Methods flowchart

The EVITA 1.1 framework has been designed and validated in the following four steps: (1) development of the provisional framework (EVITA 1.0), (2) validation framework for mental health, (3) validation through in-depth interviews, and (4) revision and finalization of the framework (EVITA 1.1). Figure 1 shows the methods in a flowchart.

Little guidance exists on how to design and validate frameworks. A detailed interactive, multidirectional process of framework validation, including identification of domains and clustering of constructs, has been described by Cane *et al.* (2012). Ward *et al.* (2009) reviewed 28 models and developed a conceptual framework for the knowledge transfer process into practice in four stages. Likewise, the SPIRIT framework was validated in sequential steps (Redman *et al.*, 2015). For the development of EVITA 1.1, we will use a similar, combined approach, following a literature review, identifying recurring themes, aggregating themes to produce a conceptual framework and validating through in-depth interviews (IDIs).

The EVITA framework has been designed through a process of co-production, iteration and validation, with version 1.0 being the input into this article and version 1.1 the output of this article.

### Development of the provisional framework (EVITA 1.0)

From our earlier systematic review of theories on research and policy interrelationships in LMICs (Votruba *et al.*, 2018), we identified seven core themes, which we synthesized and based on which we drafted a provisional framework. We added theoretical elements from leading policy process theories for agenda setting (Sabatier and Weible, 2014) and combined a policy problem with a policy solution and events in the politics sphere (Baumgartner *et al.*, 2006; Shiffman, 2007; Kingdon, 2014) with a specific framework for global health priority setting (Shiffman and Smith, 2007), which has been applied to mental health (Tomlinson and Lund, 2012).

### Development of, and validation through, a framework for mental health

Throughout the development of the framework and the IDIs, we validated the provisional framework EVITA 1.0 for its specific validity for, and applicability to, mental health. We developed a framework to assess the criteria against mental health as a policy issue and to ensure a focus on policy priority setting.

### Validation through in-depth interviews

In August and September 2018, we conducted expert IDIs to evaluate the framework. We used purposive sampling to identify 22 expert representatives of relevant groups with expertise on evidence and policy interrelations and invited them. Based on experience, we expected this number to be sufficient for data saturation. Ahead of the interview, all participants were sent a self-explanatory information pack with the provisional framework EVITA 1.0 and guiding questions. The aim was to confirm the relevance of the essential components, to validate the mechanisms and to explore their views on the practicability, effectiveness and actionability of the draft framework.

We designed, piloted and refined the protocol for IDIs (Castillo-Montoya, 2016; see Supplementary data 2). Semi-structured IDIs with experts were conducted via phone, Skype and in person by N.V.. Each interview started with open-ended questions on their overall experience on research and policy interrelations, and the overall suitability of the provisional framework EVITA 1.0, structure, components and mechanisms. Then, we more specifically assessed the validity, asking for: (1) intelligibility, (2) functionality, (3) relevance, (4) applicability (to confirm actionability) and (5) effectiveness of targeting policy agenda setting. We designed a simple framework for coding and evaluating frameworks’ components and mechanisms (see Table 1).

**Table 1:** Simple framework for evaluating frameworks

Criteria	Question that can be asked
1. Applicable and actionable	What do you think about the applicability and actionability of EVITA?
2. Functional	How do you see the elements, processes and overall EVITA framework working?
3. Intelligible	What do you think about the intelligibility of the elements and processes?
4. Relevant	What do you think about the relevance of the components? Would you use EVITA in your work? How do you see yourself using it?
5. Useful in targeting the problem	What are your thoughts regarding the focus on targeting the agenda setting stage to improve evidence–policy interrelationships for mental health in LMICs? Do you find the approach useful?

The interviews were conducted by a researcher experienced in qualitative methods (N.V.), in person, or via phone, Skype or Zoom. The interviews lasted on average one hour and were digitally recorded, deidentified, coded and analysed, applying deductive analysis and framework analysis to identify themes and verify key components, mechanisms and potential issues.

### Revision and finalization of the framework (EVITA 1.1)

We assessed the provisional conceptual framework (EVITA 1.0) to comply with our criteria (useful, practical, effective, actionable, agenda-setting focus), revised it in an iterative process and integrated the deductive analyses from the interviews. The revisions led to the finalized EVTA 1.1 framework.

## Results

### The provisional EVITA framework (EVITA 1.0)

From our earlier systematic review (Votruba *et al.*, 2018), we derived seven core themes of evidence and policy interrelations for mental health and LMICs: (1) actors, (2) capacity, (3) catalysts, (4) evidence, (5) external influences, (6) links and intermediaries and (7) political context. From these, we built the basic structure for the provisional EVITA 1.0 framework, represented in a fluid, two-stage model.

### Validation for mental health policy priority setting

EVITA 1.1 is specifically designed to support mental health becoming a policy issue. To ensure this, we developed a new validation framework for mental health policy issue priority setting, based on an earlier framework on factors of mental health policy priority (Bird *et al.*, 2011), into which we integrated specific characteristics of mental health as a policy issue (Mackenzie, 2014).

In this validation framework, we have identified these combined factors, which we grouped into four categories with 17 subcategories. Validation trough this framework clarifies how the consolidated EVITA 1.1 framework addresses all factors that hinder effective priority setting for mental health (see Table 2).

**Table 2:** Combined framework for mental health policy issue priority setting

Factors influencing mental health policy priority setting		How EVITA considers/addresses the issue
Category	Factor	
1. Cross-cutting issues	Heterogeneity	Evidence generators providing clear, specific evidence problems and solutions Capacity building Communication, relationship and partnership building Framing
	Stigma	Capacity building Communication, relationship and partnership building Framing Engaging with external context Engaging with (en)actors
2. Legitimacy of the problem	Appreciation of prevalence of problem	Framing of evidence problem Capacity building Communication, relationship and partnership building Intermediaries Engagement towards encouraging political will, motives and opportunities
	Understanding of severity of problem	Evidence generators providing clear, specific evidence on severity Capacity building Communication, relationship and partnership building Framing
	Poor media coverage	Engaging with external context Using catalysts Intermediaries
	Lack of data	Framing of evidence problem Capacity building Partnerships with (en)actors
	Under-diagnosis	Framing of evidence problem Capacity building Partnerships with (en)actors
3. Feasibility of response	Knowledge of appropriate interventions	Evidence generators providing clear solutions and interventions Framing of evidence solution Capacity building Partnerships with (en)actors
	Individualized nature of treatment	Evidence generators providing clear solutions and interventions Framing of evidence solution Capacity building Partnerships with (en)actors
	Socio-cultural beliefs on causes and treatment	Capacity building Communication, relationship and partnership building Framing Engaging with political context and external context
	Role of the informal sector	Strengthening communication, relationships and partnership building with (en)actors
	Lack of funding/low financial investment	Capacity building Communication, relationship and partnership building Framing Engaging with political context and external context Using catalysts
4. Support for response	Competing development and health priorities	Evidence generators providing clear, integrated, cross-sectoral solutions Framing of evidence solution Capacity building Partnerships with external context (funders) and political context
	Lack of advocacy	Strengthening communication, relationships and partnership building with (en)actors Advocacy Coalitions
	Collective agency of the service user	Strengthening communication, relationships and partnership building with (en)actors Advocacy Coalitions
	International commitments and engagement	Framing the mental health issue along External context: Sustainable Develpment Goals (SDGs), World Health Organisation (WHO) Mental health Action plan, etc. Partnerships with external context (funders)
	Effectiveness of networks	Strengthening communication, relationships and partnership building with (en)actors Advocacy coalitions Intermediaries Capacity building

### Outcomes of the expert consultation

We invited 22 experts from different backgrounds in academia, international organizations, development sector and policymaking, which had different focus areas on the topic, across research and practical engagement in knowledge translation, evidence-based policymaking, implementation science, mental health policymaking and LMICs.

A provisional framework EVITA 1.0 was the basic framework for the expert interviews. Thirteen expert IDIs were conducted between August and November 2018 (59% response rate). One expert did not return the consent form, so we had a final response rate of 57% and 12 completed interviews (see Table 3 for overview of the expert IDIs). Saturation was achieved within the sample.

**Table 3:** Overview of the expert IDIs

Sector	Area of work of interviewee	Number of interviews
Development organization	Researcher and expert on research/policy interrelationships (LMIC)	2
National government	Policymaker (mental health/LMIC)	1
National government	Policymaker; researcher engaging in research/policy interrelationships	1
University	Researcher on research/policy interrelationships	3
University	Researcher on research/policy interrelationships (LMIC); engaging in research/policy interrelationships (mental health/LMIC)	1
University	Researcher (mental health/LMIC); engaging in research/policy interrelationships (mental health/LMIC)	1
University	Researcher in implementation science; engaging in research/policy interrelationships	1
World Health Organisation	Policymaker (mental health/LMIC)	2
Total		12

### Validation of components and mechanisms

In the IDIs, we validated overall structure, components and mechanisms of the provisional EVITA 1.0 framework. Table 4 gives an overview of key components and mechanisms identified and validated in the interviews (column 1), the definition we applied in EVITA (column 2), the number of interviews that discussed each of these components or mechanisms in depth and qualified them as particularly essential for research–policy interrelationships (column 3) and a summary of key issues that came up in the in-depth interviews (column 4).

**Table 4:** Overview of core components and mechanisms identified and validated in the interviews

Component/mechanism	Definition applied in EVITA 1.1	Number of IDIs discussed in depth	Key issues that came up in the in-depth interviews
Core components			
Advocacy coalitions	Advocacy coalitions are knowledge communities and networks based on the same values, willing to agree on a common advocacy issue and seeking to translate their beliefs into governmental action programmes.	4	Several comments were made stressing challenges for mental health and the importance of ‘advocacy coalitions’ and ‘building networks’ for addressing these and the problems for mental health. As a result, ‘advocacy coalitions’ were added as a new core component in EVITA 1.1. *“Dialogue is a critical element. The challenge is to unify as one common voice for the issue. Instead, for mental health currently we have a dispersion of stakeholders”* (researcher in knowledge translation, LMIC, IDI5).
(En)actors	(En)actors are people and organizations engaged in mental health research, policy, practice or implementation and relevant to the process of evidence into policy agenda setting. This includes researchers from other fields, service user groups, carer/family organizations, doctors, nurses, service providers, NGOs, donors, funders, policy elites, corporate/pharmaceutical lobby groups, religious leaders, faith groups, trade unions, media, implementers and other experts.	9	Strengthening mental health service users (in treatment and recovered) to inform the conversation was raised. The role of the media has been found to be very influential and the relationship needing to be actively managed. *“Policymakers do pay attention to the public opinion. Therefore, the role of the media is very important, they need to be managed”* (researcher, LMIC, working in mental health policy context, IDI8).
Evidence generators	Evidence generators as the scientific research environment, which is systematically investigating and building verifiable scientific evidence and data. This includes organizations, people, mechanisms and the research evidence itself, which is considered relevant for use and application in policy agenda setting.	10	The qualities of the researcher and research, such as trust, reputation and being timely, were stressed by many interviewees. It was also suggested that they should go beyond providing evidence to include explanations for policymakers to make sense of their data. *“Elected people should make informed choices, and the role of the researcher is to provide this, but beyond that make them pay attention and provide understanding, by presenting and framing the issue in a convincing way and language. (…) The key is to create a common understanding of very different contextual knowledge, for instance, what we make out of figures.”* (researcher on research/policy interrelationships, IDI13). Also, the relevance of mental health research to the needs of the policymaking realities was stressed: *“Policymakers make decisions on what are important and easy solutions. (…) Often mental health research is insufficient or irrelevant to policy. The solutions provided are variable, complex, complicated, and not quickly visible.”* (policymaker, WHO, IDI4).
External influences	External influences as are the socioeconomic context, culture, societal values and beliefs relevant to forces and impulses on the issue, from outside policymaking (political context) or evidence generator sphere.	8	The external context and influences have been described as very important in relation to both mental health and LMICs. *“The external environment is extremely important for mental health, and often determines what is policy-relevant.”* (policymaker, WHO, IDI4).
Intermediaries	Intermediaries are people, organizations and structures that are engaged at the intersection of research and policy and actively facilitating the evidence and policy interrelationships. They act as knowledge brokers and agents of change, trying to linking ‘evidence generators’ and all (en)actors into a strong coalition.	8	Intermediaries have been confirmed as relevant and central by many interviewees. As relevant characteristics for intermediaries were named credibility and trustworthiness. However, there was a lack of clarity and agreement as to where these are exactly located, and as to what their role would be, ranging from sheer linkage function to leading opinions, and guiding and uniting the issue under ‘one single vision’. They were also seen as being able to facilitate the process through tenacity: *“Consider keeping the issue on the micro-policy agenda of intermediaries!”* (researcher on research and policy interrelationships, IDI3).
Political context	Political context is as the sum of national politics, policy and polity structures, institutions, mechanisms and policymaking processes. This includes power distribution, (in)formal rules, political will, interests, motives and opportunities of people and organizations involved.	10	Interactions of ‘evidence generators’ and the ‘political context’ have been described by several respondents as strongly depending on the willingness, needs and motivations of the policymakers. *“Policymakers will only listen to your problem if they have decided it’s a problem in the first place.”* (researcher on research and policy interrelationships, IDI13). The respondents also stressed the timely, yet rather unpredictable need of policymakers to access research evidence. *“And one day they will call you and say, 'I need a briefing on this, can you come over this afternoon?!' And then you need to be prepared to brief them!”* (policymaker, IDI7). It was emphasized by many interviewees that evidence generators need to understand the political context, including political hierarchies, structures, budgets and dependencies of and incentives for policymakers. *“You need to clarify the motivation for Policymakers: What is in for them? What is the evidence, what are the costs, what capacity do they have, what political pressures exists?”* (researcher in implementation science, IDI10). The interviewees also stressed the attention to clarifying the different ‘political contexts’ in LMICs. *“The political context in LMICs is very diverse and needs to be individually captured”* (researcher, LMIC, working in mental health policy context, IDI8).
Mechanisms			
Capacity	Capacity is the potential, knowledge and skills within the research–policy system and its members, to translate, uptake, engage with and use research evidence.	7	Capacity has been pointed out as an important process for facilitating research and policy relationships and recommended to be built on three levels, the individual, organizational and within the overall context. *“You don’t know what you don’t know. Therefore, it is important to increase capacity, also for implementation.”* (researcher in implementation science, IDI10). *“It is necessary to build capacity on three levels, the individual, organisational, and within the overall context”* (expert on research and policy interrelationships in LMIC, IDI11). Actionability of the provisional framework EVITA 1.0 has been found as rather limited.
Catalysts	Catalysts are prompts that enable, facilitate and trigger the uptake and use of research evidence in policy agenda setting	4	Catalysts have been confirmed throughout the interviews as mechanisms for increasing the probability of research being picked up in policy and practice. They were also seen as a key enabler for ‘political will’, i.e. increasing motivation and opportunities of policymakers to take action: *“Political will seems to relate to a personal character, but essentially this asks the questions, when do policymakers have motives and opportunities to act?”* (researcher on research/policy interrelationships, IDI13). Also, (en)actors, such as donors, have been pointed out as influential ‘catalysts’ for research evidence. *“Donors have a critical role in contributing as catalysts, through funding of research and implementation”* (researcher in implementation science, IDI10). Using the media to generate support and demand from the public and other stakeholders has also been highlighted. *“Creating community demand is a turbo-charger for catalysts, for instance using mass media and social media.”* (policymaker, IDI7).
Communication, relationship and partnership building	Communication, relationship- and partnership building are the sum of activities of strategic, intentional and long-term communication and interpersonal relationships and dialogue with other ‘(en)actors’ and the wider policy network.	6	Throughout the interviews, long-term personal relationships and collaboration in the political context were highlighted as important factors by all IDIs. In addition, ‘communication’ was stressed as important mechanisms for evidence generators aiming for policy impact. *“It is crucial to meet the right people and to be able to convince them in their language.”* (researcher on research/policy interrelationships, IDI12). Communication, relationship and network building, therefore, were added as a new mechanism. The overall framework focus shifted from evidence–policy relationships towards networks and co-creation of research and policy together with (en)actors and intermediaries and qualifying scientific research evidence of evidence generators as only one contribution within the overall knowledge ecosystem. “*The logical and narrow deduction of research does not simply translate to policymaking. Research evidence is only a small element in the entire policy decision-making process. It may only take a supporting role of policy and other actors, i.e. making policy research-guided.”* (policymaker, WHO, IDI4).
Framing	Framing is a dynamic process used by ‘evidence generators’, ‘(en)actors’, ‘intermediaries’ and ‘advocacy coalitions’ to present a topic while giving meaning, sense and interpretation through other social, psychological and cultural concepts and principles.	4	Framing of research evidence was pointed out by several interviewees as a very important element in the research to policy process. Specifically, this referred to researchers helping to strengthen the relevance and motivation for policymakers. “*The key for policymakers in taking the evidence up is that they understand and appreciate the issue. They need a personal and emotional hook.”* (policymaker, IDI7). However, the way evidence should be framed was seen as dependent on context and personal preference. *“The criteria differ for people for what they regard as important, or what is easier to be picked up. Some people prefer statistics on DALYs, others YLDs or QALYs.”* (researcher on research/policy interrelationships, IDI13).

The IDIs were open-ended, and while all components were validated, the in-depth discussions depended on the specific research/practice interest and expertise of the interviewee. We did not assess a hierarchy of components/mechanisms. The expert IDIs substantially validated EVITA 1.0’s structure, components and mechanisms. The IDIs were very helpful for evaluating the overall framework, weighing the relevance of the components and clarifying the mechanisms. Some relationships were slightly changed, and new components and mechanisms were integrated.

### Validation according to the assessment criteria

We evaluated the EVITA 1.0 framework according to our five assessment criteria: applicability/actionability, functionality, intelligibility, relevance and usefulness in targeting agenda setting (see Table 5 for outcome of the assessment criteria).

**Table 5:** Framework outcome of the assessment criteria

Criteria	Question asked	Number of IDIs approved (out of 12, *x* = *n*)
1. Actionability/applicability	What do you think about the applicability and actionability of EVITA?	4
2. Functionality	How do you see the elements, processes and overall EVITA framework working?	10
3. Intelligibility	What do you think about the intelligibility of the elements and processes?	8
4. Relevance	What do you think about the relevance of the components? Would you use EVITA in your work? How do you see yourself using it?	12
5. Usefulness in targeting agenda setting	What are your thoughts regarding the focus on targeting the agenda setting stage to improve evidence–policy interrelationships for mental health in LMICs? Do you find the approach useful?	12

Overall, the IDIs validated EVITA 1.0 according to our five assessment criteria. All interviewees confirmed that EVITA’s overall structure, components and mechanisms are relevant (*n* = 12). Most interviewees found the framework functional (*n* = 10) and intelligible (*n* = 8), and helpful input was considered on how to make mechanisms and design more intelligible and functional. All participants (*n* = 12) confirmed that targeting the agenda setting stage of the policy process was a useful addition to the framework and that it should be helpful in improving mental health research and policy interrelationships in LMICs. Only most interviewees confirmed that EVITA 1.1 provided conceptual clarity and a clear purpose, but only four (*n* = 4) participants found the provisional EVITA 1.0 framework to be actionable. Most interviewees were unsure that EVITA 1.1 was able to explain how individuals move from intention to actual behaviour change and whether it was therefore useful to develop and test interventions.

### The validated EVITA framework (EVITA 1.1)

**Figure 2:**
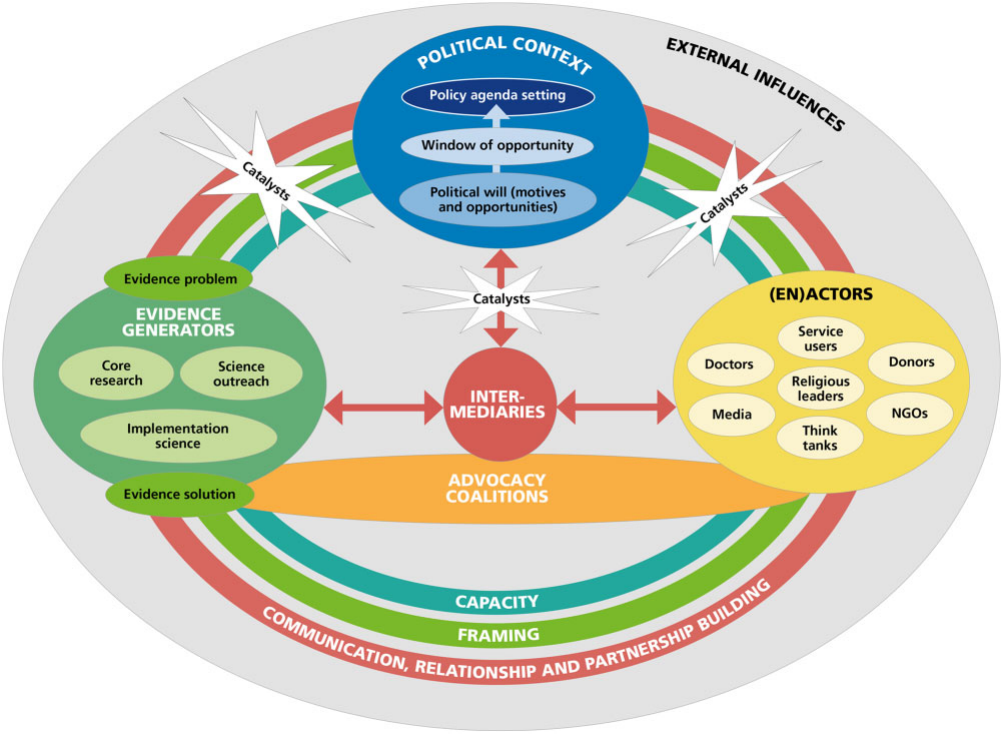
The validated EVITA 1.1 framework

In response to the interviews we revised the provisional EVITA 1.0 framework and to address the identified relevance of targeting motivations of policymakers and the influence of advocacy coalitions, we combined it with work on advocacy and influencing (Hann *et al.*, 2015; Smith and Shiffman, 2016; Shiffman *et al.*, 2016b) and advocacy coalitions (Sabatier, 1988; Jenkins-Smith *et al.*, 2014; Walt and Gilson, 2014). We are referring to this validated framework as the ‘EVITA framework (EVITA 1.1)’. We understand research–policy agenda setting as a non-linear process and envisage that EVITA 1.1 can be entered at any point. The final validated EVITA 1.1 framework (see Figure 2) consists of six core components [advocacy coalitions, (en)actors, evidence generators, external influences, intermediaries and political context] and four mechanisms (capacity, catalysts, communication/relationship/partnership building and framing). A more detailed description of EVITA’s core components and mechanisms is found in Supplementary data 3.

### Application of framework

The IDIs have rated EVITA 1.1 to be largely descriptive in its current form. In a very simplified application, we assume that the EVITA framework could describe an idealized process of improved research evidence and policy interrelationships as follows: research evidence is presented as a problem and solution and framed to the context of a specific mental health issue and LMIC setting; external influences are considered and supportive. Relationships with (en)actors and intermediaries are built up strategically. Advocacy coalitions are created; open communication and trust are established, particularly with the political context. Intermediaries establish a central connection between research and policy to facilitate the exchange of needs and promotion of the issue; (en)actors are advocating individually and through advocacy coalitions in the political and external context. research evidence from multiple directions is fed into and taken up within the political context; capacity is built up in research, policy and externally, to understand needs, relevance and options, and competencies are established; direct and in-direct engagement with research, and research evidence is co-created according to relevance and needs. Clear and strong catalysts occur and are being instigated, shaped and used effectively. Policy windows start to open up. The research topic is taken on the policy agenda.

We have illustrated how EVITA 1.1 could be applied to a case study of research evidence and policymaking in mental health care in Viet Nam (Harpham and Tuan, 2006; see Supplementary data 4).

## Discussion

This article presents the development of the EVITA 1.1 framework for mental health research and policy interrelationships in LMICs. The framework is based on a systematic review and has been validated for mental health application, and through IDIs. Four major changes resulted from the IDIs and shaped the current version of EVITA 1.1:

### Changes resulting from the interviews

The key learnings that we gained from our interviews in relation to the EVITA 1.0 framework we presented were that (1) research–policy interrelationships and knowledge translation are influenced more than initially conceptualized by other stakeholders and the entire knowledge ecosystem; (2) good communication and building long-term relationships and partnerships are even more critical than we assumed; (3) building advocacy coalitions should be added as a strategic element; and (4) EVITA needed to be more clearly actionable.

### Focus on the knowledge ecosystem

Research evidence is only one of the many factors informing policy, oftentimes rather supporting policy and other actors, rather than leading the policymaking process. The interviews stressed the influence of other ‘(en)actors’ contributions, and the relevance of building stronger links with them. Policymaking draws on a wide range of information, from think tanks, NGOs, international organizations, expert advice, public opinion, media influences, political and personal ideologies and economic, legislative and policy options and constraints (Redman *et al.*, 2015). The contributions of knowledge brokers and intermediaries to facilitate research–policy exchange have been widely stressed (van Kammen *et al.*, 2006; Bornbaum *et al.*, 2015; Haynes *et al.*, 2018; Nugroho *et al.*, 2018). EVITA 1.1 aims to embrace the knowledge ecosystem, by focusing on co-creation and collaboration with all stakeholders. Interdisciplinary, integrative approaches can be helpful to facilitate policy action for the ‘wicked problem’ mental health, through co-analysis and co-production, as for instance proposed by trans-disciplinarity/convergence science (Eyre *et al.*, 2017). In addition, EVITA 1.1 employs capacity building across the knowledge ecosystem, which has been found to be a key mechanism for long-term changes in mental health and policy in LMICs (Saraceno and Saxena, 2004; Razzouk *et al.*, 2010; Semrau *et al.*, 2018).

### Communication, relationship and partnership building

All interviewees strongly emphasized the importance of good, clear communication, creating long-term, trusted relationships and dedicated partnerships. The social, political and economic relevance of networks, norms and trust has been coined as ‘social capital’ (Halpern, 2005). Social capital is pre-existent; communication, relationship and partnerships build on it, and it is a prerequisite for establishing ‘advocacy coalitions’. A study in Uganda found that involving all relevant stakeholders throughout the process, starting at setting the research agenda to policy development and implementation, is a key factor in improving knowledge translation (Orem *et al.*, 2012). Infra-structural problems are common barriers in LMICs, but partnerships can help overcome some technical and structural barriers of research evidence access and uptake, for instance through platforms that provide access to scientific literature at little or no cost (Malla *et al.*, 2018). Communication is a key factor to increase evidence use in policy, contributing to improving the understanding of evidence and awareness for existing policy problems (Oliver *et al.*, 2014). Managing media relationships is crucial (Jacobs and Johnson, 2007; Meurk *et al.*, 2015), yet to influence agenda setting, the impact needs to aim beyond mere communication successes (Georgalakis, 2018).

### Building advocacy coalitions

We found that frameworks for mental health research–policy interrelationships rarely considered network dynamics and coalitions, despite their potential impact (Votruba *et al.*, 2018). The interviews confirmed the relevance of coalitions, so we added advocacy coalitions to the framework. Their aim is to strengthen research interrelationships with ‘(en)actors’, thus improving cohesion of the overall knowledge ecosystem, and to unify a network that leads in formulating co-developed purposively targeted ‘policy asks’. The influence of advocacy coalitions on policy has been captured in the advocacy coalition framework (Sabatier, 1988). Global health networks can help target high-burden health problems in LMICs and effectively shape global agendas (Shiffman *et al.*, 2016a). Depending on governance, leadership and composition, norms, funding and opponents within the policy environment, advocacy efforts vary in their success (Hafner and Shiffman, 2013). In addition, issues are more likely to be effective in health networks, if they are being perceived as ‘severe’ (e.g. associated with higher socioeconomic costs), as having politically uncontroversial solutions (tractability), and if the groups affected are identifiable, viewed sympathetically and able to advocate for themselves (Shiffman *et al.*, 2016b). Taking into account the far-reaching and limiting effects of stigma and discrimination (Thornicroft *et al.*, 2016), a focus of coalitions should be on including service user and carer groups and strengthening anti-stigma and awareness raising efforts (Hann *et al.*, 2015).

### Actionability

Actionable frameworks are much needed to provide a pragmatic approach to target decision-making (Redman *et al.*, 2015; Votruba *et al.*, 2018). Rarely frameworks on research and policy interrelations are giving guidance, and a structured, pragmatic approach to overcoming persisting barriers is needed (Theobald *et al.*, 2009). A key aim for the EVITA 1.1 framework is to instigate actionable behaviour. As the interviews found EVITA 1.1 to be of limited actionability, further empirical testing and improvement of actionability is needed to investigate and define specific action steps.

#### What is new and special about EVITA 1.1?

EVITA 1.1 is novel and innovative for several reasons:

1. It has a ‘novel aim’: the EVITA 1.1 framework is the first framework aiming to improve mental health research evidence translation into policy in LMICs using a focus on policy priority setting.
2. It addresses a ‘specific gap’: the EVITA 1.1 framework consolidates frameworks previously developed for research evidence translation and policy for mental health in LMICs, together with frameworks for mental health policy priority setting.
3. It is based on a ‘specific methodology’ to this aim: EVITA 1.1 was specifically developed for the purpose to improve mental health research and policy interrelationships in LMICs. A novel and unique methodology combining several steps was developed, based on a systematic review of existing frameworks. The development of the new framework was then based on components and mechanisms derived from identified frameworks, which were combined with frameworks on policy priority setting and validated for specific mental health criteria and through expert interviews.
4. It applies an ‘innovative combination of effective elements’:

- It uses ‘policy agenda setting’ as a mechanism for research impact.
- In addition, EVITA 1.1 includes a combination of effective elements, such as ‘capacity’ and ‘advocacy coalitions’, to strengthen and increase research impact in policy and practice.
- Furthermore, the embedding of the framework within the ‘knowledge ecosystem’ and a ‘central role of intermediaries’ integrates relevant network elements, which are strengthened through ‘social mechanisms’ such as ‘communication and relationship building’.
- ‘Framing’ is used as a specific mechanism to counter stigma and increase research impact on the policy agenda.

### EVITA 1.1 in relation to other frameworks

The EVITA 1.1 framework was informed both conceptually and empirically by earlier work on knowledge translation and exchange, priority setting in mental health in LMICs and health network research. A very comprehensive interfaces and receptors model has been developed by Hanney *et al.* (2003) to improve the utilization of health research in policymaking. Although not having a focus on mental health priority setting or LMIC contexts, this model represents fundamental conceptual work for health research uptake in policy. EVITA however takes an actionable approach with a focus on interrelationships and collaborative mechanisms outside the research–policy field, and a focus on policy priority setting. The definition of policy agenda setting applied in EVITA 1.1 (see Introduction section) builds on earlier research. Frameworks have been developed to understand why, how and which issues get on the policy agenda (Reich, 1995; Baumgartner *et al.*, 2006; Shiffman, 2007; Walt and Gilson, 2014). Tomlinson and Lund (2012) applied the Shiffman and Smith framework (Shiffman and Smith, 2007) to explain the low priority setting in global mental health (Tomlinson and Lund, 2012). EVITA 1.1 also integrated Kingdon’s ‘policy streams’ model consisting of problem stream, policy stream and politics stream, which are influenced by policy entrepreneurs, to create windows of opportunity (Kingdon, 2014).

Excellent other frameworks have captured the health research and policy process and developed a structured approach to increasing research uptake and very elaborate tools to support action strategies (Redman *et al.*, 2015; Makkar *et al.*, 2016a,b; Brennan *et al.*, 2017). While EVITA 1.1 currently lacks their level of actionability, EVITA’s focus is on interrelationships and continuous engagement activities with all stakeholder groups, beyond policy and research, to increase policy priority setting. EVITA also brings in the specific element of framing as a key mechanism for increasing mental health research impact in policy, which is not, or only implicitly, addressed by many other frameworks (Court and Young, 2006; Jones *et al.*, 2013). Shiffman *et al.*’s framework on the emergence and effectiveness of global health networks stressed interrelationships between stakeholders, policy issue and policy environment, including clear strategies to shape policy and public health outcomes. While this framework is focusing on health networks, EVITA shares the focus on networks, interrelationships and framing mechanisms to support policy priority setting. A study in four African countries has identified nine reasons for the low priority of mental health (Bird *et al.*, 2011). As a policy issue, mental health has been characterized with heterogeneity, stigma, lack of agency of the service user, lack of data, under-diagnosis, individualized nature of treatment, low financial investment, underrepresented role of the informal sector and lack of international engagement and of effectiveness of networks (Mackenzie, 2014).

A lack of availability of good quality, locally relevant research has been found to be a major barrier to research uptake in LMICs (Edwards *et al.*, 2019), while also power, politics and political will critically influence the knowledge exchange and agenda-setting process (Shiffman *et al.*, 2016b; Malla *et al.*, 2018). Framing strategies, such as pre-packaged and publicized policy solutions that address an identifiable and quantifiable problem, have been found to be most likely effective (Whiteford *et al.*, 2016). The impact of ‘highly effective advocates’, high-profile and credible public figures for the use of evidence has been pointed out in the youth mental health reform (Whiteford *et al.*, 2016). Others have found champions as a major factor for influencing knowledge translation and agenda setting (Harpham and Tuan, 2006; Bird *et al.*, 2011). EVITA 1.1 integrates this through the catalyst component.

Mental health shares similar contextual features with other health areas, such as HIV/AIDS, malaria or cancer, and can draw on findings on research and policy interrelationships, albeit considering contextual differences (Howell *et al.*, 2017). A study on policy priority setting in breast and cervical cancer in Ghana found that scientific and economic evidence matters, but that interpretation affects what type and how much influence research has (Reichenbach, 2002). EVITA’s communication, relationship and partnership building and capacity building mechanisms aim at reducing misconceptions and stigma and promoting mutual understanding.

### Strengths and limitations

With a very specific and narrow focus, the EVITA 1.1 framework aims to increase research uptake and impact in policy. While this is EVITA’s particular strength, it equally brings a number of limitations with it, which we address below.

#### Specific focus on scientific research aiming at the policy agenda

EVITA 1.1 is a framework aiming to enhance research and policy interrelationships in contexts with no or limited policy attention for mental health. EVITA’s key focus is on a specific part of the knowledge exchange process, limiting it to scientific research aiming at the policy agenda. EVITA does not focus on other forms of evidence but acknowledges their relevance and different strengths, weaknesses and unique power dynamics within the policy process (Jones *et al.*, 2013) and aims to integrate these through interaction with ‘(en)actors’ and embeddedness within the wider knowledge ecosystem (Koon *et al.*, 2013; Aljeesh and Khaldi, 2014).

A further limitation is EVITA’s focus on the policy agenda setting process, but not on policy implementation into practice. EVITA recognizes the relevance of policy implementation, through interrelationships with (en)actors. However, EVITA 1.1 is specifically conceptualized for countries where mental health research is barely or not at all on the policy agenda and, thus, specifically targets this priority setting stage.

#### Knowledge translation and research impact

EVITA’s scope is limited as it aims to reflect a simplified model of highly complex, non-linear processes of research translation and policymaking. Both research translation and policy processes involve many people at different levels, which take a long time, as do building relationships and establishing reputation and trust. EVITA therefore stresses the need for a long-term view for engagement in research–policy interrelationships. Further and a more general limitation is that the logical, narrow deduction of research does not directly translate into policymaking. While research efforts may be able to influence the policy process, scientific evidence is only a small part of all considerations in policymaking (Carden, 2009), and impact may, despite all efforts, not occur. To mitigate this, EVITA 1.1 envisages keeping the issue on the micro-policy agenda of advocacy coalitions and using periods where visible changes/catalysts are missing, for preparing future pushes for policy impact.

Overall, the EVITA 1.1 framework aims to give researchers and advocacy coalitions pragmatic, clear access points, without having to study the entire political and policy dynamics. Any theoretical model is only a limited reflection of a complex, unpredictable reality, but it serves to make hypothesized relationships visible, as a first step for empirical testing and refinement (Redman *et al.*, 2015).

### Expected use and impact

Interventions based on underpinning theories or conceptual frameworks have been found to be more effective (Webb *et al.*, 2010). EVITA 1.1 aims to provide such a conceptual approach for improving the interrelations of research evidence and policymaking for mental health in LMICs, and thus, contributing to reducing the excessive treatment gap for people with mental disorders.

EVITA 1.1 intends to illuminate mechanisms and stakeholders relevant in mental health research and policy interrelationships. Despite EVITA’s focus on scientific research, it can be used not only by researchers but really any stakeholder engaging to increase the use of evidence in policy, such as knowledge brokers, NGOs, patient and carer groups, families, media, public, advocacy organizations or donors. Ensuring that good quality research is being used in policymaking is not solely a responsibility of researchers. Knowledge translation and exchange are time-intensive activities and require specific skills, adding to researchers’ generally high work load and career demands including publishing peer-reviewed research. A (stronger) policy impact work force within academia is needed and shared responsibilities with different experts, such as implementation scientists and knowledge translation specialists, who are emerging research–policy backbones with a clearer understanding of, and links to, the political context and applied research.

We envisage EVITA 1.1 both as a descriptive tool and one to guide action: EVITA could be used to start the conversation and strengthen relationships between researchers and policymakers; it could be a ‘tool for change’, facilitating the research–policy dialogue for more effective use of research; or an advocacy tool for anyone in the mental health research and policy environment. Through its collaborative and co-productive mechanisms, we hope that EVITA 1.1 contributes to strengthening the availability of quality research relevant to local contexts, which is a key facilitator to knowledge translation in LMICs (Edwards *et al.*, 2019).

Beyond its very specific target group and aim, EVITA 1.1 could potentially also be relevant for application to other health areas, stigmatized conditions and inequalities. Application by this would need to be confirmed after theoretical and/or empirical testing and validation for the specific area.

In a next step, EVITA 1.1 requires empirical testing in LMICs. We will validate EVITA for contextual and user group relevance, assess relevance and interplay of mechanisms and components, refine directions and entry points and improve actionability.

## Conclusion

This article presents the new EVITA 1.1 framework, specifically designed for improving mental health research and policy interrelationships in LMICs, which uses a novel, innovative focus on policy agenda setting as a vehicle to facilitate research uptake in policy.

We developed EVITA 1.1 based on a systematic review, validated it against a newly designed framework for mental health policy priority setting, and through expert interviews. EVITA is innovative because of several distinctive features: it has a specific focus on scientific research evidence and policy interrelationships; it targets the policy agenda setting stage; it places itself within the knowledge ecosystem, integrating research, policy, (en-)actors and advocacy coalitions; and it uses communication, relationship and network building, framing and capacity building as vital and sustainable mechanisms. Next, EVITA will be validated in an LMIC case study.

## Supplementary data

Supplementary data are available at *Health Policy and Planning* online.

## Availability of data and material

All data generated or analysed during this study are included in this published article (and its supplementary data files).

## Supplementary Material

czz179_Supplementary_Data
