# Supplementary material for: The EVITA framework for evidence-based mental health policy agenda setting in low- and middle-income countries
Source: Health Policy Plan. 2020 Feb 10;35(4):424–39. doi: 10.1093/heapol/czz179 (PMC7195852; doi:10.1093/heapol/czz179)
Supplement: czz179_Supplementary_Data [file czz179_supplementary_data.zip › czz179-Suppl_Data/Supplementary data 3_Detailed components & mechanisms.docx]

**Supplementary data: Detailed background to EVITA 1.1 components and mechanisms**

This is a description of the definitions and detailed concepts of components and mechanisms we are applying in EVITA 1.1, defined after validation.

**Components**

*Advocacy Coalitions*

Why they are relevant:

Advocacy coalitions can be powerful in shaping the policy discourse. The aim is to overcome the challenge of dispersion of stakeholders and to unify one common voice for the issue. Therefore, a key element in the research-policy agenda process is to establish relationships and advocacy coalitions (Shiffman et al., 2016, Sabatier, 1987). These coalitions are based on co-production and co-development and are a substantial element of achieving increased impact of research in policy.

What can be done:

Advocacy coalitions efforts can be set up and implemented strategically. The aim for the advocacy coalition is for researchers and (en)actors to achieve a uniform voice and policy ask, common ground of values, policy aims and implementation through negotiation. With (en)actors where values are different, approaches can often still be aligned through working on common policy and implementation aims. Alliances can sometimes help to overcome and deal with obstructive enactors to prevent them from hindering the process.

*(En)actors*

Who they are:

(En)actors are part of the evidence ecosystem/ knowledge communities (Järvi et al., 2018), beyond evidence generators and policymakers. They are researchers from other fields, service user groups, carer/family organisations, doctors, nurses, service providers, NGOs, donours/funders, policy elites, the media, corporate/ pharma lobby groups, religious leaders, faith groups, trade unions, implementers, and other experts. (En)actors can be in line with the values and research and policy aims, and thus highly supportive collaborators, or they can have partly different values, research or policy aims, where they can be challenging to collaborate with, or even actively obstructive and hinder the research-policy interrelationships and agenda setting process.

What can be done:

Essential for policy agenda setting is to identify who the (en)actors are, what capacity they hold and to evaluate their power (see Shiffman & Smith 2007). Stakeholder mapping helps to clarify their level of influence, values, beliefs, links, trustworthiness, and whether they are supportive or opposing the issue, and more generally towards the research evidence/policy agenda proposal. It is also helpful to identify actors who are opposing the issue and are competing for policy impact and funding.

Activists, community and media are important drivers for policy engagement and policymaking, both as (en)actors and also as ‘links & intermediaries’. Particularly the influential role of the media requires active management of ‘relationships’. In LMICs the media are frequently controlled, and the role of bloggers emerged with a new relevance. Mental health service users (in treatment and recovered) are part of the enactors, and it is important to strengthen their role to inform the conversation.

*Evidence generators*

The qualities of the evidence:

Evidence generators provide evidence. In order to be convincing for policy agenda setting, evidence needs to fulfil crucial requirements: It has to be of good quality, rigorous, and trustworthy science, which is up to date, timely and relevant. Research needs to be clear, understandable, and accessible to policy and public (open-access, and published in non-scientific media, rather than hidden behind paywalls, in libraries or scientific conference papers), and it needs to be generalisable and applicable to local, regional or national policies. Evidence does not need to be novel but may well be a whole/part of a body of (existing) evidence. However, in LMICs’ usually low evidence base, the evidence may not easily be accessible (e.g. on prevalence) and/or systems may be lacking or incomplete to obtain the data.

The qualities of the researcher:

In evidence-policy interrelationships, specific qualities of the researcher are essential, and even more how they are being perceived by others. Only if the researcher is credible, renowned in the field and policy, trustworthy and reliable, the findings have optimal chances of being considered in the evidence-policy exchange.

Research evidence within the evidence eco-system:

We understand research evidence as one part of the overall evidence eco-system (Järvi et al., 2018), and it is only a small element in the policy decision-making process. The evidence eco-system itself is divided into different parties with their own goals and strategies. In EVITA we assume a disaggregation of the ‘evidence generators’, of core, deep ‘scientific evidence production’, ‘applied scientists’ (implementation science/ knowledge translation) and wider ‘science outreach’ and academic public and policy engagement (such as universities’ policy outreach centres). Applied scientists are looking at how to make the science work, often work multidisciplinary and address different questions than core science. Applied scientists may act as sub-intermediaries in the framing process. Wider academic public and policy engagement, such as universities’ policy outreach centres though internal politics (branding) may be similar, their aims, approaches and impact can differ greatly (e.g. compare UCL and University of Manchester). Whilst in our understanding evidence refers specifically to scientific evidence, in the framing process and particularly within ‘advocacy coalitions’, it can be useful considering additional non-research evidence.

The specific need and use of evidence in the political context:

It differs how and what policymakers perceive as ‘evidence’, and when and how they use it (for conceptual, instrumental, tactical/symbolic, imposed purposes (Hanney et al., 2003)). For instance, frequently evidence is used by policymakers instrumentally as a power/competitive resource. For the research end it is relevant to identify how and where research fits into these purposes, and finding the right timing.

Mental health research is often perceived as insufficient or irrelevant to policy, because solutions are variable, complex, complicated, not quickly visible. To overcome this challenge, it is necessary to clarify the specifics of the problem/research, and how these relate to other evidence, existing policies and budgets, and working out links of the issue with cross-cutting issues. This can help policymakers which research to choose and how to prioritise research and action, and to estimate the costs in relation to existing budgets.

*External influences*

Why it is relevant:

External influences and (local) context are the cultural and socio-economic environment and ideological setting in which research-policy interrelationships are occurring (Court and Young, 2006). External influences are crucial for a policy issue, and often determine what topic is policy-relevant. They include the culture of evidence use and the public perception of research in general, and of the specific mental health issue to be made a policy priority. It also refers to how the issue is being portrayed/avoided by the media, and the level of stigma. External influences include global policies supporting the wider issue (such as the United Nations Sustainable Development Goals), global and national donors’ strategies, priorities, and funding mechanisms.

What can be done:

Ideally, an enabling environment exists for research policy interrelationships, but oftentimes the challenge is to actively design these as much as possible through ‘communication, networking and framing’. This includes external influences through attitudes and perceptions of mental health, mental disorders, or the perception of psychology and psychiatry. For instance, in Eastern Europe, the former Soviet Union, and South America, particularly during times of dictatorship, psychiatry has been abused as instrument for political oppression, discreditation and abuse. This has had a lasting negative imprint on the perception of mental illness and the professions providing treatment and research.

*Intermediaries*

Who they are:

Intermediaries can be organisations (e.g. foundations) or individuals, such as knowledge brokers, commissioners of research (e.g. the UK NHS Chief Information Officer), established policy advisory boards (e.g. the UK All Party Parliamentary Group on Mental Health (APPGMH)), dedicated research-policy networks, committees or events, or co-production partnerships, for instance such as research project/programme partnerships. Even boundary objects (such as websites, forms, reports, twitter?) can serve as link and intermediary. Intermediaries are fluid, meaning ‘evidence generators’, policymakers and others people from the ‘political context’, as well as other ‘(en)actors’ can at times act as links and intermediaries.

Intermediaries require similar qualities as researchers, such as being trustworthy, reliable, renowned, but they have more flexibility and freedom as they are not (or less) bound by either of the academic or policy spheres and their rules. In addition, they need to master the quality of ‘communication, relationship buildin’g and establishing lasting connections.

What their role is:

Intermediaries influence how evidence is being translated, taken up and used, and they have a central role in the EVITA framework. Intermediaries are crucial in facilitating the understanding and communication between the different life realities and cultures of research and policy. Six functions of knowledge intermediaries have been identified as informing, linking, match-making, engaging, collaborating, building adaptive capacity (Jones et al., 2013).

What can be done:

As a first step, it is relevant to identify who and where these links and intermediaries lie, or who could act in this position, and whether any linkage and exchange platforms are already in place. In addition, it needs to be clarified what their influence is, how they are linked and function, and how to access or engage with them. Once relationships with intermediaries are established, trusted and efficient, their support role can expand into linking advocacy coalitions, to increase policy impact through their single vision and stronger political voice.

For researchers, intermediaries are a very important links for supporting the research to policy agenda setting process. Taking up the role of an intermediary, or establishing relationships with them, requires investment in time or money.

New ways to communicate and disseminate evidence have emerged, through social media platforms such as twitter, facebook, blogs, vlogs, and others, and they act not only as a means of ‘communication and relationship building’, but also as important links and intermediaries to policy and public. Active management of evidence and how it is being communicated through these channels is increasingly important.

Political context

Understanding the process of policymaking:

Political context is messy and fluid, and the process and nature of policymaking is chaotic and at times moving very fast. Political context and decision-making differ across cultural and country contexts, as does their capacity to absorb change (Jones et al., 2013). These processes are driven by power decisions, in many LMICs working top down, or in participatory cultures at least partly bottom up (Nash et al., 2006). For impact on the policy agenda, it is essential to understand the (in-)formal processes, rules and underlying values, as well as who the relevant people involved, such as policymakers, high-/mid-level bureaucrats, local and regional (‘street level’) bureaucrats, policy elites.

Clarifying political will, motives and opportunities:

The next stage is to identify the policy interest in research and ‘political will’. Policymakers’ will, ‘motives and opportunities’ to take action are crucial for policy agenda setting (Kingdon, 2014). Before any evidence can reach the policy context, policymakers first need to understand and appreciate the magnitude of the problem. Policymakers use evidence selectively. They need to be clear on what is in for them, in terms of evidence, costs, capacity and political pressures. The research needs to clarify the motivation for policymakers to take action, and if the evidence feeds into their arguments, it can be used as a ‘catalyst’. Policymakers pay attention to public opinion, and often make decisions on solutions that are regarded as important but also easily implementable. It is also important to understand which other acute, pressing policy issues are potentially competing with the issue.

Possible stages of policy interest in research are: (1) clear demand in government for research; (2) government interest without leadership; (3) government interest but capacity shortfall; (4) new/emerging issue activates research, but leaves policy uninterested; (5) policymakers not interested in/hostile towards research (Carden, 2009).

Recognising the policy window:

Once policymakers recognise a problem and identify a viable, politically correct and feasible solution, and have the motivation and opportunity to act on it, a so-called ‘policy window’ opens. We are applying the concept of a ‘policy window’ from Kingdon (Kingdon, 2014), and understand it as an opportunity for policy agenda setting and eventually policy change. Policy windows can be triggered by ‘catalysts’ and are critical opportunities for research and policy interrelationships. Due to the fast-paced nature and quickly changing topics in the policy environment, policy windows usually close again quickly.

Setting the policy agenda:

Thinking in policy timeframes, research timeframes differ from these substantially except for rather rare cases of commissioned research. However, the artificial construct of the policy cycle can help elucidate recurring phases of the policy process. The first dimension of policy impact, ‘framing debates and agenda setting’, has been highlighted as a key lever of power, as it is the phase where new issues gain attention (Jones and Villar, 2008). The overall aim is to create political consensus for the research topic. This ideally results in an attitudinal change, which in theory is then followed by discursive commitments, procedural change, policy content, and eventually behaviour change.

Policy agendas may be flexible, but in addition they are usually limited by (previous) budgets. Budget processes are often obscure and based on historical plans. Prioritising research is a key challenge for policymakers, as this adds a layer to existing/new budgets. Therefore, for any impact on policy agenda setting, in addition to the policy cycle, it is important to understand and consider the limitations, opportunities and timings of the budget cycle.

**Mechanisms**

*Capacity*

What it is:

Researchers and policymakers’ skills, knowledge and needs are substantially different. The capacity building process aims at understanding the different needs of policy makers and researchers, and at creating resources to fulfil them. The capacity of policymakers and bureaucrats is how far they are able to access, understand and appraise research, to plan for mental health systems and services. The capacity of researchers are their knowledge and skills, whether they are able, trained and incentivised to engage in research to policy interrelations. Capacity includes the level and quality of trusted relationships established (formal and informal) between researchers, policymakers, intermediaries and (en)actors. Capacity comprises systems in place to support dissemination to policymaking (access to journals, knowledge exchange platforms) (Semrau et al., 2018) (The Mental Health and Poverty Project, 2010). It ranges from service planners and policymakers, researchers, mental health (en)actors (such as service users and caregivers), organisational structures (universities, hospital) that support evidence to policy interrelations, and organisational policies that support and even require research provision and use in policy/program agenda setting.

Building capacity:

Capacity building aims at increasing mutual understanding, and at increasing the capacity to implement it on their own end. Capacity building focuses on enhancing connections through a number of interactions, such as information exchange workshops and forums. Capacity building can be exchanges or placement for researchers in policy and vice versa, to experience the other world. It also includes additional support and training for push efforts, user pull, and exchange for policymakers and researchers, such as co-production, creating, or even problem-definition phases where researchers, policymakers and ‘(en)actors’, in particular the media and the public engage together. Actions for capacity building can be taken on three levels: individual, organisation, context level. Strategically building up systems to train, inform, and exchange. Approaches for planning capacity building activities can include a capabilities/skills matrix, needs assessments, situational analysis, systematic reviews, qualitative interviews and stakeholder meetings, as well as the application of previous theory, evidence and experience.

Catalysts

Triggers for change:

Catalysts can make a big difference and trigger political will and the policy agenda setting process. They are mostly unplanned events, political or policy changes, a new policy or organisational demand for research, new or existing significant research findings that emerge with policy relevance, external programmes, health challenges or crises that require new research (Redman et al., 2015).

Yet, they can also be planned, as actionable behavioural impulses, or occurring as champions/VIPs. Also, donors have a critical role in contributing as catalysts through funding of research and implementation. Creating community demand is a turbo-charge for catalysts, in particular through mass media and social media.

*Communication, relationship- and partnership-building*

Why it is relevant:

A central process in EVITA is communication, relationship and partnership building. The role of the researcher is to provide up to date, accurate information to enable elected people to make informed choices. Beyond that it also implies the responsibility to raise policymakers’ awareness, and understand, important issues, by presenting these in a convincing way, language, and to ‘frame’ them to their specific problems and solutions. The role of the policymaker is to access, understand and use the evidence.

Strengthening relationships:

Developing and maintaining influential relationships between research and the political context over time is a crucial mechanism (Haynes et al., 2018). Relationships, partnerships and networks play a significant role in evidence policy processes (and are not to be confused with short-term communications campaigns). Building successful research and policy relationships requires not only meeting the right people, establishing trust and proving reliability, but also the capacity of being able to understand and convince them ‘in their language’. For strong relationships it is essential to create a common understanding of different contextual knowledge (e.g. how to read/interpret data/ figures).

Strategic communication:

Communication is an underlying key strategy of the EVITA framework. It aims at finding out what needs to change for new ideas to be explored and to develop consensus to instigate action. Communication is key to facilitate that an issue is being picked up by policymakers. It can be used strategically by researchers to feed in to change peoples’ perceptions of the research issue. Communication can also be strategically used by advocacy coalitions as a ‘pincer movement’, on different levels of the policy environment. This requires determining ‘who’ in the coalition (researcher/ mental health council/ associations etc.) talk about ‘what’ (burden of disease/ societal impact/ financial burden on health system/ donour funding and other external opportunities/ etc.) and ‘in which way’ (data/ research/ survivor experiences/ other evidence etc.) to ‘whom’ in the policy sphere (chief of staff/ member of staff/ key person in administration).

Building lasting partnerships

Once trustworthy, reliable relationships have been established and supported by effective communication, lasting partnerships can be established. They support mutual understanding, reliability, knowledge exchange and research uptake. Partnership building targets a substantial, long-term and goal-oriented collaboration between ‘evidence generators’, ‘policy’ and other ‘(en)actors’. Partnerships can arise around a specific research/policy collaboration, or as a general knowledge exchange facilitator platform, but they contribute substantially towards integrating and linking knowledge communities within the wider knowledge ecosystem (Lavis et al., 2006).

*Framing*

Why it is relevant:

Policymakers need to understand why they should use the specific evidence. Framing of the evidence clarifies the relevance and incentive for policymakers (Koon et al., 2016). In order to be priority, evidence needs to create a sense of urgency. ‘Create this sense of urgency, or jump on the bandwagon.’ (policymaker, IDI7)

How to do it:

Framing starts with formulating a policy question. Evidence is most effective in policymaking when it is very specific evidence, which (a) meets an existing policy ‘question/problem’ (is there a need for the research?); (b) is able to provide a ‘solution’ to this problem (can something be done about it?); and (c) is ‘feasible for action’ though policy (is the solution capable to be implemented and have impact)?

Framing means identifying the status quo in relation to the issue, and in what way the new evidence diverges from this status quo, and then adapting the evidence to the context and policy question. It is helpful to understand how the evidence/issue is currently being perceived (e.g. strong stigma and negative perceptions of people with mental illness), and to frame it avoiding extreme breaks with current findings (unless they are very positive/cost effective/beneficial). Alternative forms of evidence that are used and valued by policy and the public need to be considered, and how the new evidence fits best within these. It is helpful to identify ongoing projects and funding where mental health can fit in, and link with ‘advocacy coalitions’ around broader issues. Framing includes shaping the ideas, discourse and the language used in relation to mental health and mental illness. Benchmarking and ranking can function as strong drivers for resource allocation (mental health vs. other issues). However, criteria differ for what people regard as important (e.g. emotional frames or economic costs), and what they find easier to pick up (e.g. the concepts of YLL (Years of Life Lost) vs. QALYs (Quality-Adjusted Life Year) and DALYs (Disability-Adjusted Life Year)), and thus need to be tested and adapted.
