# Supplementary material for: The EVITA framework for evidence-based mental health policy agenda setting in low- and middle-income countries
Source: Health Policy Plan. 2020 Feb 10;35(4):424–39. doi: 10.1093/heapol/czz179 (PMC7195852; doi:10.1093/heapol/czz179)
Supplement: czz179_Supplementary_Data [file czz179_supplementary_data.zip › czz179-Suppl_Data/Supplementary data 4_EVITA in action.docx]

**Supplementary data 4: EVITA in Action**

To illustrate how EVITA could be applied to a case study, we used a report of research evidence and policymaking in mental health care in Viet Nam (Harpham & Tuan, 2015):

- An NGO presented data to Parliament, they were not being perceived as ‘advancing its own agenda’ (ie. reliable, trustworthy, objective).
- The NGO described mental illness as a poverty-related issue, and this dovetailed with the senators’ agenda in a one-day meeting (capacity & relationship building).
- Shortly after they followed up with solutions and engaged with other external influencers (media).
- Focus on clear issue (depression) and cross-sectoral links (mothers, poverty, education).
- Research converged with the priorities of policy department enabled incorporation into long-term plans.
- A senior policy actor championed it (catalyst).
- Research gained the attention of policy-makers because the researchers engaged with key stakeholders at an early stage of their research, the data were regarded as rigorous and the timing of the release of the data was opportune in that it coincided with a 5-year planning cycle.
