# Supplementary material for: The EVITA framework for evidence-based mental health policy agenda setting in low- and middle-income countries
Source: Health Policy Plan. 2020 Feb 10;35(4):424–39. doi: 10.1093/heapol/czz179 (PMC7195852; doi:10.1093/heapol/czz179)
Supplement: czz179_Supplementary_Data [file czz179_supplementary_data.zip › czz179-Suppl_Data/Supplementary data 2_Protocol for expert interviews.docx]

**Supplementary Data 2:** **Protocol for expert interviews on the preliminary framework**

| **ID No.** |  |
| --- | --- |
| **Date** |  |
| **Time** |  |
| **Place** |  |
| **Interviewer** |  |
| **Interviewee** |  |
| **Group** |  |
| **Title** |  |

1. **Opening & Consent**

- Thank you for taking time to give your feedback on our framework.
- I’ve emailed you the presentation outline of the framework, the questions, the information sheet and the consent form.
- If you are happy with it, can you please sign/ send me your signature on the consent form?
- For the interview, the plan is that I will briefly ask about your background, what works in evidence-policy process, then your perception of the overall framework, and your views of its components.
- **Would it be helpful as a start, if I gave you a bit of background?**
- I am doing a PhD on how to get mental health evidence into policy. And the aim is to design an action framework to improve mental health research evidence translation into policymaking in low- and middle-income countries.
- We have performed a systematic review of actionable theories, frameworks and models of research-policy interrelationships, where we identified four frameworks that were applicable to mental health, LMICs and actionable, but none had a focus on agenda setting.
- We performed synthesis of these four frameworks, and designed a new preliminary framework with these elements, and in order to more effectively target policymaking, we also added the central element of policy agenda setting. To improve research impact on policy priority setting.
- We have invited a number of experts working in the context of research evidence and policy interrelations, to comment on our draft framework of research-policy interrelations for mental health in LMICs
- The aim of this interview is to get your expertise and to assess process validity, as well as practicability, effectiveness, and actionability of the draft framework.
- Do you have any questions so far, or are you happy for me to go ahead with the questions?

1. **Interview**
   1. **Your role and background**

- Can you say a bit on your role and how you relate/ have related with the research and/or policy process?
  1. **What works in research-policy exchange**
- Generally, from your experience, what works/ has worked in getting research evidence into policy? What is important to facilitate the use of research in policymaking?
  1. **The EVITA framework**
- Overall, what are your thoughts on our framework?
- Do you think the processes between the components are displayed as realistically?

1. **Intelligibility**: What do you think about the intelligibility of the elements and processes?
2. **Functionality**: How do you see the elements, processes and overall EVITA framework working?
3. **Relevance:** What do you think about the relevance of the components? Would you use EVITA in your work? How do you see yourself using it?
4. **Applicability & actionability:** What do you think about the applicability and actionability of EVITA?
5. **Targeting policy agenda-setting**: What are your thoughts regarding the focus on targeting the agenda setting stage to improve evidence-policy interrelationships for mental health in LMICs?

- Which processes do you feel might be most effective? And which most challenging?
- Do you feel we have missed anything relevant?

1. **Closing and thank you**

- This was very useful. Thank you very much for giving your time and expertise to help designing our framework.
- With your comments we will now revise the framework and test it in different case studies for my PhD. I will get back to you with updates and our findings as soon as possible.

1. **Notes**

The key points you raised will go into the revision of the framework:
