# Supplementary figures and images for: The EVITA framework for evidence-based mental health policy agenda setting in low- and middle-income countries
Source: Health Policy Plan. 2020 Feb 10;35(4):424–39. doi: 10.1093/heapol/czz179 (PMC7195852; doi:10.1093/heapol/czz179)

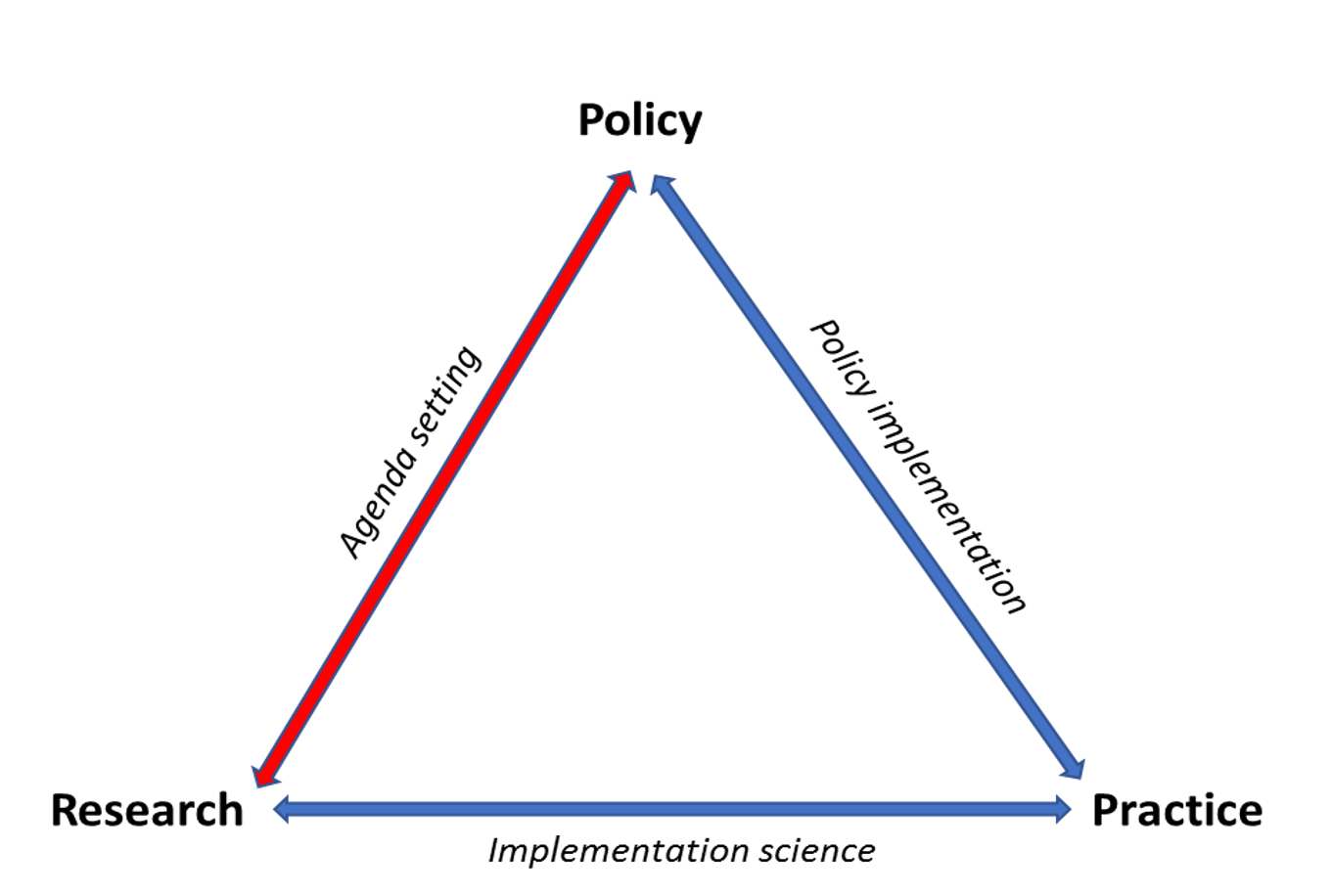

Supplement: czz179_Supplementary_Data [file czz179_supplementary_data.zip › czz179-Suppl_Data/Supplementary data 1_Figure SD1 Simplified evidence–policy–practice model.tif]
